# Leading Risk Factors for Congenital Deafness in the Context of Universal Neonatal Screening: Our Observations in a Four-Year Retrospective Study

**DOI:** 10.3390/ijns10010011

**Published:** 2024-01-30

**Authors:** Antoine Paul, Fanny Bense, Claire Boithias Guerot, Sofia De La Rubia, Cécile Lebeaux, Jean-François Papon

**Affiliations:** 1 Otolaryngology Department—Cochlear Implant Center, Hôpital Bicêtre, Assistance Publique—Hôpitaux de Paris, Paris-Saclay University, 94270 Kremlin-Bicêtre, France; 2 Neonatal Intensive Care Unit Department, Hôpital Bicêtre, Assistance Publique—Hôpitaux de Paris, Paris-Saclay University, 94270 Kremlin-Bicêtre, France; 3 Neonatal Intensive Care Unit Department, Hôpital Antoine Béclère, Assistance Publique—Hôpitaux de Paris, Paris-Saclay University, 92140 Clamart, France; 4 Center of Perinatal Care of Val-de-Marne Department, 94000 Créteil, France

**Keywords:** screening, hearing, infant, congenital deafness, genetic

## Abstract

It has been demonstrated that universal hearing neonatal screening (UHNS) is efficient at providing early diagnosis and rehabilitation for deafness. The risk factors of deafness in children have been identified, but less specifically in those diagnosed after UHNS. In this study, we aim to study these factors in infants who were referred after screening and to compare our experience to recent data. We studied infants referred to our department for diagnosis after screening between January 2018 and December 2021. Their medical history and neonatal hearing risk factors were assessed. Associations between factors were also analyzed. Sixty-six infants were included. A family history of deafness (47%), syndromic deafness (41%), intrauterine growth retardation or prematurity (19.7%), and prolonged NICU admission (18%) were the most observed factors. When analyzing according to these associations, family history of deafness and syndromic cases remained the most prevalent factors (74%), while only five cases (7.8%) presented with other neonatal risk factors only. The majority of congenital hearing loss cases are observed in infants with suspected genetic deafness. Parental counseling, the diagnostic pathway, as well as the healthcare system should be adapted according to these risk factors.

## 1. Introduction

The universal newborn hearing screening (UNHS) implemented in past decades in developed countries aims to screen for congenital deafness as one of the most prevalent sensorial congenital conditions. One out of every one thousand livebirths is the prevalence rate commonly communicated to the general population to understand the importance of this screening; however, the frequency is variable depending on cohort size, methods of screening, and the type of hearing loss (sensorineural and/or conductive) included. Studies before UNHS implementation showed values around 0.8–1.8/1000 [1,2] while recent authors reported 2 to 3.4/1000 livebirths [3,4]. In addition, distinguishing permanent from transient deafness makes this estimation difficult: moderate hearing loss (HL) may lead to a completely different prognosis depending on its cause, for example, whether caused by transient otitis media effusion (OME), which is a frequent diagnosis, or by permanent sensorineural HL. Moreover, spontaneous resolution is often observed during the first year of life, which could be explained by subclinical OME [5].

This UNHS, which has been mandatory in France since 2014, is performed in maternity or the neonatal intensive care unit (NICU). However, despite the intention to avoid any delayed diagnosis by performing screening throughout the country, it is becoming increasingly difficult to follow up with all children because of staff shortages in referral centers in the face of growing demography. Our team primarily assesses infants with no response in both ears in the first month. Unilateral “REFER” results are reassessed later, considering that unilateral deafness does not prevent the development of oral language, keeping in mind not only the difficulties caused by unilateral HL that have become now well-established [6] but also the risk of bilateral evolution during this process.

The Joint Committee of Infant Hearing (JCIH) listed these neonatal hearing loss risk factors in 2019 [7]. However, practitioners must remain aware that certain causes of deafness may have become relatively more or less common over the past several decades. Medicine has improved substantially in this time, particularly in neonatal care, with better knowledge in pregnancy monitoring, delivery practice, and neonatal care. These improvements may have been more likely to alleviate certain factors than others, and the existing literature and data must be examined accordingly.

Given these medical developments, there is particular interest in studying the medical context of congenital deafness diagnosed in our referring department over the past few years, comparing them to those of recent studies in order to develop an overall understanding of the existing literature since the updated recommendations of the JCIH 2019.

This study has been set up in our ENT department located in the University Bicêtre Hospital, which belongs to the Paris-Sud-Université Paris-Saclay group, including two tertiary maternity centers associated with NICU departments (Bicêtre and Antoine Béclère Hospitals). Our department has been designated as a referral center for infants referred from both hospitals when deafness is suspected after neonatal screening. The results of screening and deafness diagnoses are sent to the local departmental perinatal care units (located in Val-de-Marne and Haut-de-Seine departments) that are charged with collecting these data and notifying lost-to-follow-up infants. They are required to send reports to the regional agency responsible for centralizing data in the Ile-De-France region (Coordination Francilienne de Dépistage Neonatal de l’Audition—CFDNA).

## 2. Materials and Methods

This is a retrospective monocentric observational study, analyzing the data of all infants presenting with permanent deafness. Only sensorineural and permanent conductive (caused by auricular dysplasia/atresia, microtia, and/or ossicle malformations) hearing loss was considered. At least 18 months of follow-up was required for inclusion in order to confirm permanent deafness with the characterization of hearing loss. For this reason, we studied cases diagnosed in a period ranging from January 2018 to December 2021, providing a minimum of a nearly two-year context window for studying this population before analysis.

As recommended by the national health authorities, screening tests should be performed a second time before discharge after an initial “REFER” result. This recommendation was followed in the maternity ward as well as the NICU when infants were transferred early in their first days of life. If no response was observed in either or both ears, they were referred to audiology centers within the following month.

During the first meeting in the outpatient clinic, the following criteria were registered by consulting maternity or NICU reports and by questioning parents about the presence of congenital abnormalities mostly associated with deafness (craniofacial, ophthalmologic, kidney), a family history of deafness, or perinatal factors such as the term of pregnancy (prematurity defined as under 37 weeks of amenorrhea), birth weight (low when under 2800 g), intrauterine growth retardation (IGR) when under the 10th weight percentile, the method of delivery, mother-to-child pre or perinatal infections, jaundice and hyperbilirubinemia +/exsanguinous-transfusion, bacterial meningitis, NICU admission, and the use of ototoxic drugs during pregnancy or in infants.

Clinical examination was performed before ABR for every infant, which included an otoscopy head and neck evaluation looking for associated craniofacial malformations (cleft palate, neck or auricular tip or tag, aural dysplasia). Other abnormalities were noted when reported by other physicians (e.g., cardiac, renal, ophthalmologic anomalies). Genetic findings were also recorded (molecular diagnosis or specific clinical syndrome). Imaging results of middle or inner ear malformations were noted when available.

An ABR was performed with the Collin Neuro-Audio Device, stimulating at a rate of 21.1 Hz with chirp alternative stimulations. The severity of deafness was represented by the threshold noted on the ABR result (lowest intensity with identifiable wave V, testing by 5 dB steps). We jointly defined the severity levels as close to the BIAP (International Bureau for AudioPhonology, www.biap.org, accessed on 10 July 2017) classification for audiometry as possible: mild deafness from 25 to 40 dB, moderate from 45 to 65 dB, severe from 70 to 85 dB and profound at 90 dB and above. Asymmetric hearing losses are specified in our results.

MRI is usually performed in those with SNHL above 8 months of age when cochlear nerves are considered as sufficiently identifiable. A CT scan is performed within the first years of life before undergoing cochlear implantation or when a permanent bilateral conductive hearing loss is diagnosed. Otherwise, CT is performed from the age of 5 years in order to detect middle or inner ear anomalies when the mastoid is sufficiently pneumatized. Biological tests are also performed to identify associated anomalies that may guide etiologic research, especially cytomegalovirus (CMV) serology and kidney, and thyroid assessment. The CMV serology was considered only when negative to exclude it as a cause. In instances where CMV maternofetal infection (cCMV) is strongly suspected, PCR on a dried blood spot could be requested. Kidney ultrasonography is usually performed in the case of syndromic deafness.

The correlation between deafness risk factors was calculated using the Pearson regression method. A *p*-value under 0.05 was considered significant.

Written and oral information was given to parents. They were informed that this retrospective study does not influence their child’s care, and they are free to decline the use of their children’s data at any moment. This study was approved by the ethical committee of the French Society of Otolaryngology (Comité Ethique Francais de l’ORL, CEF-ORL). The number reference is n°2023-10-030-AP.

## 3. Results

### 3.1. Cohort Characteristics

**Figure 1:**
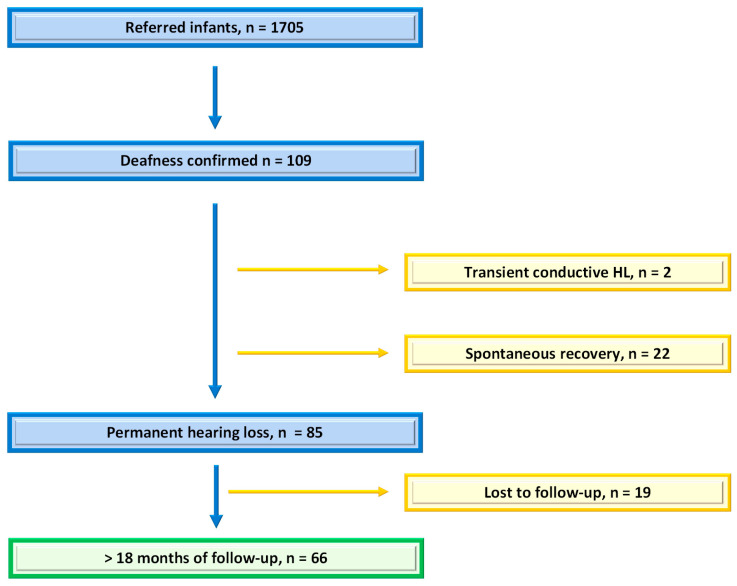
Flow chart: number of infants referred after UNHS and number of those included in the study with permanent deafness and at least 18 months of follow-up.

One thousand seven hundred and five infants were referred to our department between January 2018 and December 2021. After excluding those presenting with transient deafness corresponding to otitis media effusion, the spontaneous normalization of hearing loss without clear etiology, and infants lost to follow-up, 66 of them were included (see flow chart, Figure 1). The cohort characteristics are detailed in Table 1.

### 3.2. Risk Factors

Among the factors considered relevant to assessment, we observed the following: a family history of deafness (in first or second-degree relatives) in 31 (47%) patients, syndromic hearing loss in 27 (41%) patients, prematurity and/or intrauterine growth retardation in 13 (19.7%) patients, NICU hospitalization in 12 (18%) patients (more than half of them needed more than 5 days of ventilation or suffered from cardiopulmonary arrest), jaundice in 8 (12%) patients, CMVmi in 3 (4.5%) infants, exposure to ototoxic treatment in 2 (1%) infants and bacterial meningitis in 1 infant (1.5) (Table 2).

**Figure 2:**
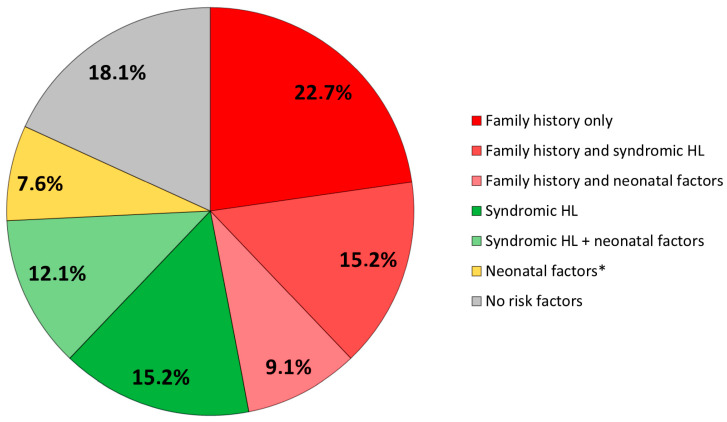
Distribution of risk factors. HL: hearing loss. * Infants presenting with neonatal risk factors without family history or syndromic HL are described in the text.

When looking at the association between these factors, we observed that the majority of our cohort presented with a family history of deafness and/or syndromic deafness (*n* = 49, 74%), whereas only five infants (7.8%) had solely neonatal risk factors (NICU hospitalization, the need for ventilatory assistance, experienced cardiopulmonary distress, were diagnosed with jaundice, cCMV, ototoxic exposure, or meningitis). Among the latter, one infant presented with premature birth, IGR, NICU hospitalization with ventilation and hyperbilirubinemia needing phototherapy, one presented with IGR, one underwent NICU hospitalization, ventilation, and ototoxic medication, one had deafness as a result of cCMV, and one presented with neonatal bacterial meningitis. Twelve children (18%) had none of the above risk factors (Figure 2). The correlations between these risk factors were analyzed, as shown in Table 3, and it was observed that a family history of deafness or syndromic cases was correlated to none of the other factors. On the other hand, correlations were observed between most neonatal factors.

### 3.3. Imaging Findings

Fifteen children (22.7%) presented with inner or middle ear malformations, with predominantly cochleovestibular malformations and/or cochlear nerve deficiency (CND) corresponding to hypoplasia or agenesia. Imaging was normal in 23 children. Imaging was pending in the 28 children left. The previously studied risk factors were also noted specifically in this subgroup of cases with ear malformations, revealing familial history and syndromic hearing loss in most cases (Table 4).

### 3.4. Genetic Causes

Genetic diagnoses were distinguished into confirmed molecular diagnosis and clinically identified syndromic deafness. Unidentified syndromic deafness was assumed to be of genetic origin. Thus, molecular diagnosis was found in 10 children, with *GJB2* mutations being the most frequent findings in three cases. Syndromic deafness was identified in 24 out of 66 children (36%), with Waardenburg and CHARGE syndromes well described in 3 and 1 cases, respectively). On the other hand, 20 children presented with abnormalities associated with deafness without any clearly identified genetic cause. Data regarding congenital abnormalities and genetic findings are shown in the Appendix A.

## 4. Discussion

UHNS was successfully implemented in our area, as during the studied period, both Val-de-Marne and Haut-de-Seine department perinatal care units reported a coverage rate of above 98% births in accordance with the JCIH objectives of 95%. UNHS has demonstrated high effectiveness in the early diagnosis of congenital deafness, lowering the age of auditory rehabilitation in developed countries [5]. Early diagnosis and rehabilitation provide better language development and, consequently, an improvement in other cognitive skills due to their mutual interactions in the context of brain plasticity during the first years of life [8].

Our recent experience highlights the large prevalence of cases that are either syndromic or associated with a family history of deafness; thus, we can assume that genetics is the main cause. This observation correlates with Shearer et al.’s overview (2017) [9], reporting that 80% of congenital deafness has a genetic cause. As most children in our cohort had not had genetic counseling yet, the prevalence could be higher after molecular testing.

A NICU admission of more than 5 days has been commonly considered a risk factor for deafness as it potentially involves hypoxemia or cardiopulmonary defects. Thus, the brain and, as a consequence, inner ear hypoxemia may lead to delayed maturation with the risk of auditory neuropathy [10]. In order to specifically focus on infants having suffered from hypoxemia, we noted those needing more than 5 days of invasive or non-invasive ventilation. Cardiopulmonary arrest was also considered as a relevant criterion. Similarly, jaundice during the neonatal period may affect the auditory nerve fibers in proximity to the inner hair cell synapses due to bilirubin toxicity [11].

As children may present with several risk factors, we have shown their association. Thus, it clearly appears that neonatal risk factors are rarely isolated. No significant correlation was noted between the two most observed risk factors (family history of deafness or a syndromic hearing loss) and the other factors, so we can consider them independent. On the contrary, most of the neonatal factors are significantly correlated with one another.

Our experience is consistent with the results of the recent study of Verstappen et al. (2023) [12], showing genetics as the primary cause (32.1%), followed by infectious causes (7.1%). A panel of only a few genes was used at the beginning of the period studied, while many cases of “unknown causes” had a familial history of deafness or consanguinity, assuming possible genetic causes. The principal identified risk factors were family history and hyperbilirubinemia.

- Zhou et al. (2022) shared the idea that the epidemiology of the usual risk factors studied has evolved, at least in developed countries, due to the “improvement in the quality of medical care” [3]. Four main factors were observed in their cohort as follows: craniofacial anomalies, NICU admission, family history, and advanced maternal age (AMA). However, their study dealt with fewer infants (*n* = 25), and surprisingly, the frequency of congenital conductive deafness was greater than sensorineural deafness (15 versus 8), making a comparison with our work difficult.

Fitzgibbons et al. (2021) studied the UNHS results in 613,027 newborns, taking risk factors into account and gathering 10 years of data (2007–2016) [13]. They demonstrated an association between permanent deafness and a family history of hearing loss, craniofacial anomaly syndromes, and gender. Infants presenting with risk factors are four times more likely to have permanent deafness than those without risk factors. The rate of false positives (the rate of infants referred after neonatal screening, with normal hearing after being assessed in audiology centers) observed was 44.8% (193/1034) versus 72% (4109/5701), respectively, in infants presenting with risk factors and those without.

In view of these results, it appears necessary to weigh the cost of systematic screening and the false positive rate, making the concern of cost-effectiveness legitimate. There have been few economic studies published within recent years, and it is difficult to apply economic analyses from foreign countries to France due to economic differences (the cost of exams, staff salaries, cost of hospital admission, currency value, etc.), and such work has not been undertaken in France since UNHS was implemented.

All these recent results should make us reconsider the risk hierarchy of neonatal deafness when organizing the diagnosis pathway. Systematically referring infants too early may generate useless anxiety for many parents. Moreover, deafness was diagnosed in only 5% of referred infants in our study.

We are convinced that the policy of neonatal screening must be continued to avoid any delays in rehabilitation. However, making this screening universal leads to referral centers becoming progressively overwhelmed. These centers must ensure not only that all referred infants are assessed in an appropriate timeframe but also that a suitable follow-up schedule is established according to the infant’s medical history. Thus, efforts to inform parents and reassess hearing should be spread between audiology centers and other health structures according to the medical context.

- It must also be taken into account that neonatal screening has been set up in developed countries with a suitable healthcare system, but it is hardly affordable in poor countries. Data from the recent literature should help physicians in such countries to select infants with a higher risk of congenital hearing loss and to focus their limited means on this specific population.

Our study has limitations in terms of the methodology as a retrospective work with possible bias; as a tertiary referral center, severe cases were referred to our hospital, leading to recruitment bias, especially when looking at the rate of syndromic cases. However, our results should not be too dissimilar to the local epidemiology: by simply relying on the reported numbers of livebirths in the two main maternity units during the studied period (13,794 and 14,432 livebirths in Bicêtre and Antoine Béclère hospitals, respectively), we could estimate a prevalence of 2.3/1000, which is consistent with the usual epidemiology reported in the literature.

- The limitations of current devices must also be taken into account as they have some difficulty in screening mild deafness. TEOAE devices provide the detection of outer hair cell function when a hearing threshold does not exceed 35 dBHL, whereas aABR devices validate responses at a threshold ranging from 25 to 40 dBHL [7].
- Moreover, as both aABR and OAE were not systematically performed together, we could have underestimated cases of auditory neuropathy spectrum disorders.

## 5. Conclusions

This work supplements data from the recent literature, supporting that priority should definitely be given to suspicion of genetic causes (a familial history of deafness or syndromic cases), especially when the healthcare system cannot cover the entire population. Studies should be conducted at a larger scale in France to confirm our observation, and we hope this study will be taken into account in future recommendations for screening policies.

## Figures and Tables

**Table 1:** Cohort characteristics.

	*n* (%)
**Number of infants**	**66 (100)**
Male	35 (53)
Female	31 (47)
**Delivery**	
Normal delivery	40 (60)
Cesarean	14 (21)
Unspecified	12 (19)
**Mean age of diagnosis (SD)**	**5.7 mo (3.7)**
**Mean birth weight (SD)**	**3061.9 g (789)**
Unknown	2
**Unilateral deafness**	**14 (21)**
Severity of deafness at diagnosis	
Mild	0
Moderate	5
Severe	4
Profound	5
**Bilateral deafness**	**52 (79)**
Severity of deafness at diagnosis	
Mild	3
Asymmmetrical mild/moderate	2
Moderate	14
Asymmmetrical moderate/profound	4
Severe	8
Profound	21
**Screening tests in maternity/NICU**	
TEOAE only	22 (33)
aABR only	28 (42)
Both TEOAE and aABR	11 (17)
Unknown	5 (8)

mo: month, g: gram, HL: hearing loss, NICU: neonatal intensive care unit, TEOAE: transient-evoked auditory emission, aABR: automatic auditory brainstem response.

**Table 2:** Prevalence of congenital deafness risk factors.

CONGENITAL DEAFNESS RISK FACTORS	*n* (%)
**Family history of deafness**	**31 (47)**
1st degree	28
2nd degree	3
**Syndromic forms**	**27 (41)**
**Prematurity and/or intrauterine growth retardation**	**13 (19.7)**
**NICU hospitalization**	**12 (18)**
Noninvasive ventilation > 5 days	2
Invasive ventilation > 5 days	2
Cardiopulmonary arrest	4
Mean duration of hospitalization (days)	32 (1–180)
**Jaundice**	**8 (12)**
Jaundice + phototherapy	6
Benign jaundice − no treatment	1
Hyberbilirubinemia > 350 umoL/mL and/or needing exsanguino-transfusion	1
**CMV maternofetal infection**	**3 (4.5)**
Other maternofetal infections	-
**Perinatal ototoxic treatment**	**2 (3)**
<5 days	1
>5 days	1
**Bacterial meningitis**	**1 (1.5)**

NICU: neonatal intensive care unit, CMV: cytomegalovirus.

**Table 3:** Correlation between risk factors.

	FHd	Sd	Prematurity	IGR	NICUa	Ventilation	CMVmi	HB	BM	Ode
FH	1									
SD	0.42	1								
Prematurity	0.242	0.234	1							
IGR	0.412	0.433	0.804 **	1						
NICUa	0.354	0.512	0.874 **	0.782	1					
Ventilation	0.204	0.199	0.948	0.755 *	0.889 **	1				
cCMV	−0.175	0.411	−0.267	0.005	−0.01	−0.268	1			
HB	0.387	0.055	0.872 **	0.700 *	0.735 *	0.767 **	−0.391	1		
BM	−0.273	−0.275	−0.511	−0.484	−0.517	−0.509	−0.232	−0.482	1	
Ode	0.325	0.021	0.298	0.173	0.373	0.461	−0.453	0.299	−0.481	1

Correlations were calculated using the Pearson regression method. * means with a *p*-value < 0.05, ** means with a *p*-value < 0.005. FHd: family history of deafness, Sd: syndromic deafness, IGR: intrauterine growth retardation, NICUa: neonatal intensive care unit admission. cCMV: maternofetal CMV infection, HB: hyperbilirubinemia, BM: bacterial meningitis, Ode: ototoxic drug exposure.

**Table 4:** Inner/middle ear malformations and risk factors of congenital hearing loss among them.

	*n*
**Middle/inner ear malformations**	**15**
CV anomalies	5
CV + CND	4
Cochlear nerve deficiency	2
Enlarged vestibular aqueduct	1
EVA + CV malformations	1
Gusher syndrome	1
Ossicles dysplasia	1
Normal imaging	23
Unknown	28
**TOTAL**	**66**
**Risk factors among children with ear malformations**	
Familial history	4
syndromic hearing loss	3
Family history and syndromic hearing loss	2
Syndromic hearing loss and perinatal factors	3
perinatal factors	2
No risk factors	1

CV: cochleovestibular, CND: cochlear nerve deficiency, EVA: enlarged vestibular aqueduct.

## Data Availability

Data are contained within the article.

## Appendix A. Main Malformations in Syndromic Deafness

Combinations are not specified. Several children presented with several malformations shown in this table.

**Table A1:** Main malformations in syndromic deafness.

Associated Abnormalities	*n* (%)
**Craniofacial**	**14 (21.2)**
Microtia	5
Fistula—pits	5
Hemifacial microsomia	2
Palate cleft	2
**Brain**	**8 (12.1)**
Epilepsy (west)	1
Microcephaly	1
Sacrococcygeal defects	1
Brain atresia	1
Anoxo-ischemic brain	1
Cerebellar cyst	1
Axial hypotonia—white matter anomaly	1
Intraparenchymal calcification	1
**Eye**	**4 (6)**
Coloboma	1
Iris heterochromia	3
**Heart**	**4 (6)**
Ischemic cardiomyopathy	1
Atrioventricular canal	1
Aortic hypoplasia	1
Tetralogy of Fallot	1
**Kidney**	**3**
Hypoplasia	1
Renal fusion	1
Renal insufficiency	1
**Limbs**	**3 (4.5)**
Spastic equinus deformity	
Polydactyly	
Talus valgus	
**Digestive tracts**	**3 (4.5)**
Oesophageal atresia	2
Digestive tract stenosis	1
**Vertebra**	**2 (3)**
Sacrococcygeal anomalies	1
Hemivertebral hypoplasia	1

**Table A2:** Genetic molecular diagnosis and syndromic deafness.

	*n* (%)
**Molecular diagnosis**	**8 (12)**
*GJB2*	3
*SLC26A4*	1
*CHD23*	1
Chromosome 6 duplication	1
*TECTA*	1
*ANKRD11* (KGB syndrome)	1
*B4GALT7*	1
*OTUD6B*	1
**Syndromic deafness**	**4 (6)**
Waardenburg syndrome	3
CHARGE syndrome	1
**Unidentified syndromic deafness**	**20**
